# Trends in the Prevalence of Hypertensive Heart Disease in China From 1990 to 2019: A Joinpoint and Age–Period–Cohort Analysis

**DOI:** 10.3389/fpubh.2022.833345

**Published:** 2022-03-16

**Authors:** Dan Xu, Jingcen Hu, Shuyu Wang, Lian Chen

**Affiliations:** ^1^Department of Geriatrics, Yuyao People's Hospital, Ningbo, China; ^2^Department of Epidemiology, Zhejiang Provincial Key Laboratory of Pathophysiology, School of Medicine, Ningbo University, Ningbo, China; ^3^Department of Cardiology, Yuyao People's Hospital, Ningbo, China

**Keywords:** prevalence, hypertensive heart disease, joinpoint regression analysis, age-period-cohort effect, trends

## Abstract

To the best of our knowledge, no comprehensive estimates of the descriptive epidemiology of and trends in hypertensive heart disease (HHD) in China have been reported. In this study, the temporal trends in the prevalence of HHD in China from 1990 to 2019 were estimated using an age–period–cohort model. Data related to HHD burden were collected from the Global Burden of Disease Study 2019. From 1990 to 2019, HHD prevalence in China showed decreasing trends in both sexes combined (average annual percentage change [AAPC]: −0.2%, 95% confidence interval (CI: −0.3% to −0.2%) and in males (AAPC: −0.5%, 95% CI: −0.5% to −0.4%), but significant increases in the age groups of 15–19, 20–24, …, and 60–64 years. The age effect analysis showed an increase in HHD prevalence from 50 to 94 years in both males and females, the period effect analysis showed a slight increase in HHD prevalence from 2009 to 2019 in females, and the cohort effect analysis showed a consistent decline in HHD prevalence from earlier to later birth cohorts in both males and females.

## Introduction

As a main complication of hypertension, hypertensive heart disease (HHD) contributes to the growing burden of cardiovascular disease worldwide (1) and is associated with an increased risk of death (2–4). Notably, HHD accounted for 7.9 million cases and 320,089 deaths in China in 2019.

An accurate national-level epidemiological study of HHD has not yet been conducted in China. The Global Burden of Disease Study 2019 (GBD 2019) provides a comprehensive assessment of the global burden of HHD by age, sex, and year, thereby providing a wealth of information on temporal trends in HHD prevalence in China (5).

We aimed to investigate the long-term trends in HHD prevalence in China between 1990 and 2019 by using an age–period–cohort framework to examine age-, period-, and cohort-specific effects by sex. Our findings provide evidence-based information for the development of HHD prevention strategies.

## Materials and Methods

### Overview

The GBD 2019 provides comprehensive global estimates of the prevalence, risk factors, and years lived with disability for 369 diseases and injuries from 1990 to 2019 (5). To evaluate disease/injury prevalence, the GDB 2019 used DisMod-MR 2.1, a Bayesian meta-regression tool that has a compartmental model structure with a variety of differential equations that combine sparse and heterogeneous epidemiological data (5). HHD prevalence estimates for the global population provided by the GBD 2019 were used as references for calculating the age-standardized prevalence rate (ASPR) of HHD in China. Data are reported as estimates with 95% uncertainty intervals (UIs). The Yuyao People's Hospital reviewed and approved this study.

### Data Sources

Data related to the HHD burden were obtained from the Global Health Data Exchange (GHDx)—the online catalog of the GBD data. The GBD uses all available health data sources through comprehensive searches and summaries of published and unpublished data (5). HHD was defined according to the International Classification of Diseases, Tenth Revision codes I11.051, I11.901, and I11.951. The original data used in the GBD 2019 for estimating HHD prevalence in China were mainly obtained from the Cause of Death Reporting System of the Chinese Center for Disease Control and Prevention, the Disease Surveillance Points System, and the Maternal and Child Surveillance System, all of which are considered to be nationally representative.

## Statistical Analysis

### Joinpoint Regression Analysis

To determine the magnitude of the temporal trends in HHD prevalence, the average annual percentage changes (AAPCs) and corresponding 95% confidence intervals (CI) were calculated using joinpoint regression (6). The AAPC was calculated as a geometrically weighted average of various annual percentage change (APC) values obtained from the regression analysis (7). This analysis was performed using Joinpoint software (version 4.6.0.0) developed by the Surveillance Research Program of the US National Cancer Institute (Bethesda, MD, USA).

### Age–Period–Cohort Analysis

Age-Period-Cohort (APC) models have been adopted in epidemiology to study a wide variety of health outcomes (8). The purpose of APC models is primarily to decompose data trends into age, period and cohort effects. Age effects have been described as phenomena associated with growing older; period effects as general influences that vary through time or epochs; and cohort effects as phenomena associated with individuals born around the same time (i.e., birth cohorts).

The age-specific rates were calculated for 5-year age groups (15–19, 20–24, …, 90–94), consecutive 5-year periods from 1990 to 2019, and consecutive 5-year birth cohort groups (1904–1909, 1909–1914, …, 1999–2004) to estimate the net age-, period-, and birth cohort-specific effects on HHD prevalence, respectively.

The age–period–cohort model can be expressed as *Yj* = μ + α* age j* + β* period j* + γ* cohort j* + ε*i*, where Yj denotes the response variable, i.e., the net effect on HHD prevalence for group j; α, β, and γ denote the coefficients of age, period, and cohort of the model, respectively; μ denotes the intercept of the model; and εi denotes the residual of the model.

The age–period–cohort intrinsic estimator (APC-IE) method was used to provide coefficient estimates for age, period, and cohort effects on HHD prevalence (Supplementary Table S1), and these coefficients were converted to exponential values (exp(coef.) = e^coef.^) that denote the relative risks (RRs) of HHD prevalence across age groups, periods, and birth cohorts relative to the corresponding average risk levels (9). The age–period–cohort modeling was performed using an online tool available at: https://analysistools.cancer.gov/apc/.

## Results

### Descriptive Analysis

Supplementary Figure S1 shows the ASPR of HHD stratified by sex in the Chinese population from 1990 to 2019. The ASPR decreased during 1990–2005 and then increased slightly during 2005–2019. Specifically, it decreased from 461.02 in 1990 to 433.54 in 2019 per 100,000 persons in both sexes combined and from 475.72 in 1990 to 417.14 in 2019 per 100,000 persons in males. The annual ASPRs in males were significantly higher than those in females between 1990 and 2019.

Supplementary Table S2 presents the APCs and AAPCs in HHD prevalence by sex in the Chinese population from 1990 to 2019. The joinpoint regression results revealed that the ASPR in both sexes combined decreased significantly during 1990–2005 and increased significantly during 2005–2014 and 2017–2019, with an overall AAPC of −0.2% (−0.3, −0.2%) during 1990–2019. A similar trend was also seen in females. In males, the ASPR decreased significantly during 1990–2005 and 2012–2017 and increased significantly during 2005–2012 and 2017–2019, with an overall AAPC of −0.5% (−0.5, −0.4%) during 1990–2019. The largest increase in HHD prevalence was observed during 2017–2019 (APC: 1.8, 95% CI: 1.4 to 2.1% in both sexes combined; APC: 0.5, 95% CI: 0 to 1% in males; and APC: 2.8, 95% CI: 2.1 to 3.4% in females).

### Trends in the ASPR of HHD Based on a Joinpoint Regression Analysis

Table 1 shows the AAPCs in the ASPR of HHD in China from 1990 to 2019. HHD prevalence increased significantly in the age groups of 15–19, 20–24, …, and 60–64 years in both sexes combined, in males, and in females. HHD prevalence also increased in the age groups of 65–69 and 70–74 years in females. In addition, compared with the younger age groups (15–19 to 35–39 years), the older age groups (40–44 to 55–59 years) showed greater increases in HHD prevalence. In contrast, HHD prevalence decreased significantly in the age groups of 70–74 to 90–94 years in both sexes combined and in males.

**Table 1:** The average annual percent changes (AAPCs) in age-specifific prevalence rate of hypertensive heart disease in China, 1990–2019.

**Age group (years)**	**Prevalence (AAPC)**	**Male**	**Female**
	**Both gender**		
Age-standardized rate	−0.23^*^ (−0.38 ~−0.29)	−0.53^*^ (−0.57 ~−0.45)	0.05 (−0.16 ~ 0.09)
15–19	0.44^*^ (0.35 ~ 0.47)	0.36^*^ (0.27 ~ 0.38)	0.43^*^ (0.41 ~ 0.44)
20–24	0.23^*^ (0.21 ~ 0.38)	0.23^*^ (0.21 ~ 0.30)	0.23^*^ (0.24 ~ 0.36)
25–29	0.35^*^ (0.34 ~ 0.36)	0.37^*^ (0.24 ~ 0.38)	0.35^*^ (0.35 ~ 0.37)
30–34	0.47^*^ (0.33 ~ 0.56)	0.43^*^ (0.41 ~ 0.57)	0.46^*^ (0.37 ~ 0.57)
35–39	0.55^*^ (0.50 ~ 0.66)	0.54^*^ (0.46 ~ 0.77)	0.65^*^ (0.54 ~ 0.76)
40–44	0.65^*^ (0.60 ~ 0.70)	0.63^*^ (0.57 ~ 0.65)	0.74^*^ (0.64 ~ 0.73)
45–49	0.64^*^ (0.61 ~ 0.78)	0.64^*^ (0.56 ~ 0.67)	0.86^*^ (0.84 ~ 0.86)
50–54	0.77^*^ (0.55 ~ 0.87)	0.57^*^ (0.55 ~ 0.66)	0.92^*^ (0.84 ~ 0.94)
55–59	0.65^*^ (0.53 ~ 0.78)	0.50^*^ (0.40 ~ 0.60)	0.86^*^ (0.77 ~ 0.95)
60–64	0.37^*^ (0.27 ~ 0.39)	0.10^*^ (0.00 ~ 0.11)	0.51^*^ (0.55 ~ 0.65)
65–69	−0.24^*^ (−0.29 ~−0.17)	−0.46^*^ (−0.53 ~−0.48)	0.24^*^ (0.14 ~ 0.27)
70–74	−0.37^*^ (−0.44 ~−0.28)	−0.64^*^ (−0.77 ~−0.58)	0.17^*^ (0.04 ~ 0.19)
75–79	−0.33^*^ (−0.43 ~−0.27)	−0.78^*^ (−0.87 ~−0.68)	0.04 (0.00 ~ 0.10)
80–84	−0.47^*^ (−0.54 ~−0.37)	−0.74^*^ (−0.88 ~−0.77)	−0.22^*^ (−0.28 ~−0.10)
85–89	−0.62^*^ (−0.68 ~−0.65)	−0.75^*^ (−0.87 ~−0.74)	−0.43^*^ (−0.52 ~−0.48)
90–94	−0.67^*^ (−0.85 ~−0.59)	−0.87^*^ (−0.96 ~−0.77)	−0.67^*^ (−0.85 ~−0.56)

* *Significantly at P < 0.05*.

### Age–Period–Cohort Analysis

The estimated RRs of HHD prevalence due to age, period, and cohort effects are shown in Table 2.

**Table 2:** Hypertensive heart disease prevalence relative risk (RR) due to age, period, and cohort effects.

**Factor**	**Prevalence (RR, 95% CI)**	
	**Males**	**Females**
**Age, years**		
15–19	0.00 (0.00 ~ 0.00)	0.00 (0.00 ~ 0.00)
20–24	0.01 (0.00 ~ 0.01)	0.01 (0.00 ~ 0.01)
25–29	0.03 (0.03 ~ 0.04)	0.03 (0.03 ~ 0.03)
30–34	0.07 (0.06 ~ 0.07)	0.06 (0.05 ~ 0.06)
35–39	0.13 (0.12 ~ 0.14)	0.11 (0.10 ~ 0.11)
40–44	0.28 (0.26 ~ 0.30)	0.24 (0.23 ~ 0.25)
45–49	0.69 (0.65 ~ 0.72)	0.61 (0.58 ~ 0.63)
50–54	1.20 (1.14 ~ 1.27)	1.08 (1.04 ~ 1.12)
55–59	1.62 (1.54 ~ 1.70)	1.45 (1.40 ~ 1.50)
60–64	2.68 (2.56 ~ 2.81)	2.40 (2.32 ~ 2.48)
65–69	6.07 (5.79 ~ 6.35)	5.54 (5.37 ~ 5.73)
70–74	9.97 (9.53 ~ 10.43)	9.40 (9.10 ~ 9.70)
75–79	12.82 (12.26 ~ 13.41)	12.59 (12.20 ~ 12.99)
80–84	15.74 (15.06 ~ 16.46)	17.30 (16.77 ~ 17.85)
85–89	21.33 (20.40 ~ 22.30)	28.37 (27.50 ~ 29.27)
90–94	27.95 (26.73 ~ 29.23)	41.61 (40.33 ~ 42.94)
**Period**		
1994–1999	0.98 (0.92 ~ 1.04)	0.97 (0.93 ~ 1.02)
1999–2004	1 (0.96 ~ 1.04)	0.99 (0.96 ~ 1.02)
2004–2009	1 (1 ~ 1)	1 (1 ~ 1)
2009–2014	1.02 (0.99 ~ 1.06)	1.03 (1 ~ 1.06)
2014–2019	1 (0.95 ~ 1.06)	1.04 (1 ~ 1.09)
**Cohort**		
1904–1909	1.38 (1.01 ~ 1.9)	1.16 (1.04 ~ 1.29)
1909–1914	1.24 (1.09 ~ 1.42)	1.05 (0.98 ~ 1.13)
1914–1919	1.14 (1.04 ~ 1.24)	0.95 (0.9 ~ 1.01)
1919–1924	1.06 (0.99 ~ 1.14)	0.9 (0.85 ~ 0.95)
1924–1929	1 (0.94 ~ 1.07)	0.89 (0.85 ~ 0.93)
1929–1934	0.96 (0.9 ~ 1.02)	0.88 (0.84 ~ 0.92)
1934–1939	0.93 (0.88 ~ 0.99)	0.88 (0.84 ~ 0.93)
1939–1944	0.92 (0.88 ~ 0.97)	0.9 (0.86 ~ 0.94)
1944–1949	0.93 (0.89 ~ 0.98)	0.93 (0.89 ~ 0.97)
1949–1954	0.96 (0.91 ~ 1.01)	0.96 (0.92 ~ 1)
1954–1959	1(1 ~ 1)	1(1 ~ 1)
1959–1964	1.04 (0.98 ~ 1.1)	1.04 (0.99 ~ 1.09)
1964–1969	1.08 (1.01 ~ 1.16)	1.08 (1.02 ~ 1.14)
1969–1974	1.11 (1.01 ~ 1.22)	1.11 (1.02 ~ 1.2)
1974–1979	1.13 (0.98 ~ 1.3)	1.12 (1 ~ 1.26)
1979–1984	1.14 (0.92 ~ 1.4)	1.14 (0.96 ~ 1.37)
1984–1989	1.15 (0.87 ~ 1.53)	1.16 (0.92 ~ 1.47)
1989–1994	1.17 (0.76 ~ 1.79)	1.17 (0.83 ~ 1.67)
1994–1999	1.17 (0.46 ~ 2.99)	1.17 (0.54 ~ 2.54)
1999–2004	1.18 (0.03 ~ 42.55)	1.19 (0.06 ~ 22.11)

*RR, relative risk; CI, confidence interval*.

### Age Effect

**Figure 1:**
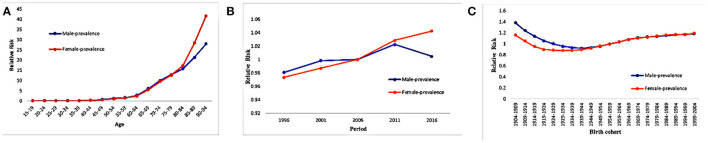
Hypertensive heart disease prevalence relative risks due to **(A)** age; **(B)** period; **(C)** cohort effects.

Controlling for period and cohort effects led to a sharp increase in the age-related RRs of HHD prevalence in females from 1.08 (95% CI: 1.04–1.12) in the age group of 50–54 years to 41.61 (95% CI: 40.33–42.94) in the age group of 90–94 years. The RRs also continuously increased with increasing age in males (Figure 1A).

### Period Effect

In females, the period effect on HHD prevalence increased slightly during 2009–2014 and 2014–2019 (Table 2, Figure 1B). In males, the period effects on HHD prevalence were relatively invariant.

### Cohort Effect

The cohort effect analysis showed that HHD prevalence continuously decreased from earlier to later birth cohorts (Table 2, Figure 1C). Specifically, from the 1904–1909 to the 1944–1949 birth cohort, the RRs of HHD prevalence significantly decreased by 30.4 and 17.2% in males and females, respectively (Table 2, Figure 1C).

## Discussion

This study used longitudinal data from the GBD 2019 to investigate the effects of age, period, and birth cohort on the temporal trends in HHD prevalence in China from 1990 to 2019. It was found that HHD prevalence increased in the age groups of 15–19, 20–24, …, and 60–64 years during 1990–2019. However, the older age groups (40–44 to 55–59 years) showed greater increases in HHD prevalence than did the younger age groups (15–19 to 35–39 years). Age–period–cohort modeling of the net age effect showed that HHD prevalence consistently increased with advancing age from 65 to 94 years in females. In addition, the period effect analysis revealed a slight increase in HHD prevalence from 2009 to 2019 in females, while the cohort effect analysis revealed a continuous decrease in HHD prevalence from earlier to later birth cohorts (from 1904–1909 to 1944–1949) in both males and females.

The joinpoint regression results showed that the ASPR of HHD decreased significantly in both sexes combined and in females during 1990–2005 and increased significantly during 2005–2014 and 2017–2019. Rapid urbanization, economic development, and demographic and epidemiological transitions in China, together with the aging of China's population, might have contributed to these increases in the ASPR of HHD since 2005 (10). The most recent study on HHD used data from the GBD 2017 to obtain the total percentage change in disease burden from 1990 to 2017, and HHD is still a major public health challenge worldwide with an increasing prevalence rate over the past decades (11).

In addition, the prevalence of hypertension, which is the leading risk factor for HHD, has increased in China since 2005. This increased detection of hypertension from 2005 onwards was due to using the reduced cutoff points (140/90 mmHg for hypertension and 120/80 mmHg for normal blood pressure) established in the 2005 edition of the Chinese Guidelines on Prevention and Control of Hypertension compared with those (160/95 mmHg for hypertension and 140/90 mmHg for normal blood pressure) used in previous national surveys (1958, 1979–1980, 1991, and 2000) (12). Thus, primary prevention, treatment, and control of hypertension should be advocated in China.

Significant increases in HHD prevalence were observed in the age groups of 15–19, 20–24, …, and 60–64 years in both sexes, in males, and in females during the observation period (1990–2019), and further increases were observed in females in the age groups of 65–69 and 70–74 years. The increases in HHD prevalence in younger age groups may be due to increases in high-risk behaviors, such as increased fat intake, reduced physical activity, and smoking, and the consequent rapid increases in the prevalence of hypertension, dyslipidemia, diabetes, and obesity that are implicated in HHD. Lifestyle modifications such as a healthy diet, smoking cessation, reduced salt intake, and physical activity should be advocated in these groups.

The age effect analysis showed that HHD prevalence consistently increased with advancing age from 65 to 94 years in females. This finding indicates that HHD prevalence significantly increased with advancing age, mainly in older people, which might be attributable to the aging population in China. Older people are also more likely to have other health complications or comorbidities, the prevalence of which also steadily increases with advancing age. For example, the prevalence of hypertension has been found to increase with age, and under the age of 50, it is more prevalent in males, whereas above the age 50, it is more prevalent in females (13). The explanation for this between-sex difference remains unclear, but it is generally acknowledged that cardiovascular disease usually occurs at a younger age in males than in females (14).

The period effect analysis showed that there was a slight increase in HHD prevalence in females during 2009–2014 and 2014–2019. In males, there was little effect of period on HHD prevalence. Period effects are usually influenced by a combination of historical events and environmental factors (15). Thus, increased life expectancy, changes in lifestyle behaviors, and rapid urbanization probably accounted for the increased prevalence of hypertension (from 13.6 in 1991 to 27.9% in 2015) (16) and further contributed to the increasing trend in HHD prevalence.

The cohort effect analysis revealed that HHD prevalence was higher in earlier birth cohorts (1904–1909) than in later birth cohorts (1944–1949). This is likely because the later birth cohorts received more education and had a better awareness of health and disease prevention than the earlier birth cohorts (17). The improvements in public health and treatment of medical conditions also partially explain the decreasing trends in HHD prevalence observed in the later birth cohorts.

This is the first study to use the latest estimates of HHD prevalence in China provided by the GBD 2019, to explore the temporal trends in HHD prevalence using the age–period–cohort model. An age–period–cohort analysis was performed to synchronously evaluate net age, period, and cohort effects on the trends in HHD prevalence (6), and the APC-IE method was used to decompose the temporal trends (9). However, the following limitations should be acknowledged. First, the GBD 2019 estimates were obtained based on a large number of sources of varying quality, and thus, the findings of the GBD 2019 must be verified by nationwide epidemiological studies. Second, age–period–cohort analysis considers community as an analytical unit, which might lead to ecological fallacies. Finally, the causal effects were not be evaluated in this study.

## Conclusion

In China, HHD prevalence showed a decreasing trend from 1990 to 2019 in both sexes combined and in males. An age effect analysis showed that HHD prevalence increased in older males and females from 2009 to 2019. In addition, a period effect analysis showed that HHD prevalence increased slightly in females during this period, while a cohort effect analysis showed that the prevalence decreased from earlier to later birth cohorts (from 1904–1909 to 1944**–**1949). These findings suggest that future HHD prevention strategies should be should be developed to target the elderly female population and the management of high blood pressure in China.

## Data Availability Statement

The original contributions presented in the study are included in the article/Supplementary Material, further inquiries can be directed to the corresponding author/s.

## Author Contributions

LC and DX performed the study, analyzed data, and wrote the manuscript. JH and SW contributed obtaining and analyzing data. LC designed the study. DX and JH analyzed data and wrote the manuscript. All authors contributed to the article and approved the submitted version.

## Funding

This work was supported by the Innovative Talent Support Plan of the Medical and Health Technology Project in Zhejiang Province (2021422878) and research fund of Yuyao People's Hospital (2019QB11).

## Conflict of Interest

The authors declare that the research was conducted in the absence of any commercial or financial relationships that could be construed as a potential conflict of interest.

## Publisher's Note

All claims expressed in this article are solely those of the authors and do not necessarily represent those of their affiliated organizations, or those of the publisher, the editors and the reviewers. Any product that may be evaluated in this article, or claim that may be made by its manufacturer, is not guaranteed or endorsed by the publisher.
