# Supplementary material for: Trends in the Prevalence of Hypertensive Heart Disease in China From 1990 to 2019: A Joinpoint and Age–Period–Cohort Analysis
Source: Front Public Health. 2022 Mar 16;10:833345. doi: 10.3389/fpubh.2022.833345 (PMC8966025; doi:10.3389/fpubh.2022.833345)
Supplement: Supplementary file 1 [file Data_Sheet_1.docx]

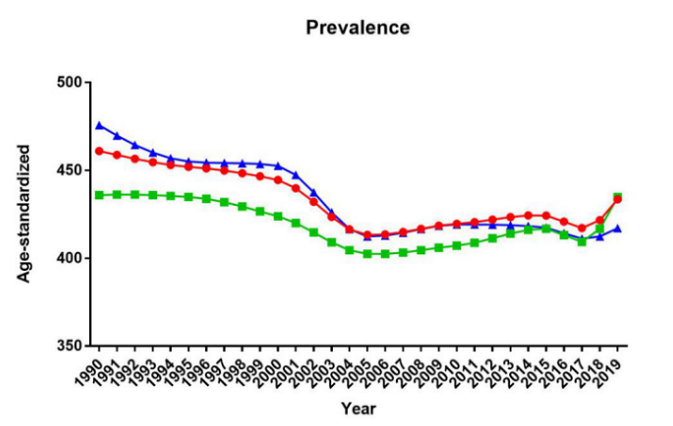


Supplementary Fig 1**-**Trends of the ASPR for Hypertensive heart disease from 1990 to 2019, at all ages. ASPR, age-standardized prevalence rate;

Supplementary Table 1 Age-period-cohort (APC) model analysis results of hypertensive heart disease prevalence rate in China, by gender.

| Variable | Prevalence (coefficient, 95% CI) | |
| --- | --- | --- |
|  | Males | Females |
| Age | | |
| 15-19 | -0.1(-0.35~0.15) | -0.13(-0.34~0.07) |
| 20-24 | -0.09(-0.32~0.13) | -0.12(-0.3~0.07) |
| 25-29 | -0.09(-0.29~0.12) | -0.1(-0.27~0.07) |
| 30-34 | -0.08(-0.26~0.1) | -0.08(-0.23~0.07) |
| 35-39 | -0.07(-0.23~0.09) | -0.06(-0.19~0.06) |
| 40-44 | -0.06(-0.2~0.07) | -0.05(-0.16~0.06) |
| 45-49 | -0.06(-0.17~0.05) | -0.03(-0.12~0.06) |
| 50-54 | -0.05(-0.14~0.04) | -0.01(-0.08~0.06) |
| 55-59 | -0.04(-0.11~0.03) | 0.01(-0.05~0.06) |
| 60-64 | -0.03(-0.09~0.02) | 0.03(-0.02~0.07) |
| 65-69 | -0.03(-0.08~0.02) | 0.04(0~0.08) |
| 70-74 | -0.02(-0.07~0.03) | 0.06(0.02~0.1) |
| 75-79 | -0.01(-0.08~0.05) | 0.08(0.02~0.13) |
| 80-84 | -0.01(-0.09~0.08) | 0.1(0.03~0.17) |
| 85-89 | 0(-0.11~0.11) | 0.11(0.03~0.2) |
| 90-94 | 0.01(-0.12~0.14) | 0.13(0.03~0.24) |
| Period | | |
| 1994-1999 | -0.02(-0.08~0.04) | -0.03(-0.07~0.02) |
| 1999-2004 | 0(-0.04~0.04) | -0.01(-0.04~0.02) |
| 2004-2009 | 0(0~0) | 0(0~0) |
| 2009-2014 | 0.02(-0.02~0.06) | 0.03(0~0.06) |
| 2014-2019 | 0(-0.05~0.06) | 0.04(0~0.08) |
| Cohort | | |
| 1904-1909 | 0.32(0.01~0.64) | 0.15(0.04~0.26) |
| 1909-1914 | 0.22(0.08~0.35) | 0.05(-0.02~0.12) |
| 1914-1919 | 0.13(0.04~0.22) | -0.05(-0.11~0.01) |
| 1919-1924 | 0.06(-0.01~0.13) | -0.1(-0.16~-0.05) |
| 1924-1929 | 0(-0.06~0.07) | -0.12(-0.17~-0.07) |
| 1929-1934 | -0.04(-0.1~0.02) | -0.13(-0.18~-0.08) |
| 1934-1939 | -0.07(-0.13~-0.01) | -0.12(-0.17~-0.08) |
| 1939-1944 | -0.08(-0.13~-0.03) | -0.11(-0.15~-0.07) |
| 1944-1949 | -0.07(-0.12~-0.02) | -0.08(-0.12~-0.03) |
| 1949-1954 | -0.04(-0.09~0.01) | -0.04(-0.08~0) |
| 1954-1959 | 0(0~0) | 0(0~0) |
| 1959-1964 | 0.04(-0.02~0.1) | 0.04(-0.01~0.09) |
| 1964-1969 | 0.08(0.01~0.15) | 0.08(0.02~0.13) |
| 1969-1974 | 0.11(0.01~0.2) | 0.1(0.02~0.18) |
| 1974-1979 | 0.12(-0.02~0.26) | 0.11(0~0.23) |
| 1979-1984 | 0.13(-0.08~0.34) | 0.13(-0.04~0.31) |
| 1984-1989 | 0.14(-0.14~0.43) | 0.15(-0.09~0.38) |
| 1989-1994 | 0.16(-0.27~0.58) | 0.16(-0.19~0.51) |
| 1994-1999 | 0.16(-0.77~1.09) | 0.16(-0.62~0.93) |
| 1999-2004 | 0.17(-3.41~3.75) | 0.18(-2.74~3.1) |

CI, confidence interval.

Supplementary Table 2**-** Trends in hypertensive heart disease prevalence rate by gender in China, 1990-2019

| Segments | Both gender | | Males | | Females | |
| --- | --- | --- | --- | --- | --- | --- |
|  | Year | APC(95% CI) | Year | APC(95% CI) | Year | APC(95% CI) |
| trend 1 | 1990-2000 | -0.36* (-0.43~-0.33) | 1990-1994 | -1.03* (-1.24~-0.95) | 1990-1998 | -0.15* (-0.26~-0.18) |
| trend 2 | 2000-2005 | -1.55* (-1.67~-1.47) | 1994-2000 | -0.16 (-0.26~0.10) | 1998-2005 | -1.16* (-1.28~-14) |
| trend 3 | 2005-2014 | 0.46* (0.37~0.47) | 2000-2005 | -1.97* (-2.16~-1.87) | 2005-2014 | 0.45* (0.37~0.57) |
| trend 4 | 2014-2017 | -0.78* (-1.04~-0.35) | 2005-2012 | 0.38* (0.33~0.44) | 2014-2017 | -0.57 (-1.16~0.18) |
| trend 5 | 2017-2019 | 1.81* (1.40~2.10) | 2012-2017 | -0.45* (-0.67~-0.39) | 2017-2019 | 2.86* (2.14~3.47) |
| trend 6 | - | - | 2017-2019 | 0.54* (0.07~1.04) | - | - |
| AAPC | 1990-2019 | -0.26* (-0.38~-0.29) | 1990-2019 | -0.57* (-0.59~-0.41) | 1990-2019 | 0.07 (-0.16~0.09) |

APC, annual percentage change; AAPC, average annual percent change; CI, confidence interval; ^*^ Significantly at P < 0.05.
